# Role of Circulating Tumor Cells (CTC), Androgen Receptor Full Length (AR-FL) and Androgen Receptor Splice Variant 7 (AR-V7) in a Prospective Cohort of Castration-Resistant Metastatic Prostate Cancer Patients

**DOI:** 10.3390/cancers11091365

**Published:** 2019-09-13

**Authors:** Carlo Cattrini, Alessandra Rubagotti, Linda Zinoli, Luigi Cerbone, Elisa Zanardi, Matteo Capaia, Paola Barboro, Francesco Boccardo

**Affiliations:** 1 Academic Unit of Medical Oncology, IRCCS San Martino Polyclinic Hospital, 16132 Genoa, Italy; carlo.cattrini@gmail.com (C.C.);; 2 Department of Internal Medicine and Medical Specialties (DIMI), School of Medicine, University of Genoa, 16132 Genoa, Italy; 3 Department of Health Sciences (DISSAL), School of Medicine, University of Genoa, 16132 Genoa, Italy

**Keywords:** AR-V7, AR-FL, castration-resistant prostate cancer, prognostic biomarkers, circulating tumor cells

## Abstract

Background: Circulating tumor cells (CTC), androgen receptor full-length (AR-FL), and androgen receptor splice variant 7 (AR-V7) are prognostic in patients (pts) with metastatic castration-resistant prostate cancer (mCRPC). AR-V7 seems to predict resistance to androgen-receptor signaling inhibitors (ARSi). Methods: We assessed the association of CTC, AR-FL, and AR-V7 with prostate-specific antigen (PSA) response and overall survival (OS). We used a modified AdnaTest CTC-based AR-FL and AR-V7 mRNA assay. Chi-square test, Fisher Exact test, Kaplan–Meier method, log-rank test, Cox proportional hazards models were used as appropriate. Results: We enrolled 39 mCRPC pts, of those 24 started a first-line treatment for mCRPC (1L subgroup) and 15 had received at least two lines for mCRPC (>2L subgroup). CTC, AR-FL, and AR-V7 were enriched in >2L compared to 1L subgroup. Detection of these biomarkers was associated with a lower percentage of biochemical responses. Only 1/7 AR-V7+ pts had a PSA response and received cabazitaxel. Median OS was 4.7 months (95% CI 0.6–8.9) in AR-V7+ pts and not reached in AR-V7− pts. AR-V7 was the only variable with prognostic significance in the Cox model. Conclusion: AR-V7, CTC, and AR-FL are associated with advanced mCRPC and AR-V7+ predicts for shorter OS.

## 1. Introduction

The treatment landscape of metastatic castration-resistant prostate cancer (mCRPC) has radically changed over recent years. Androgen-receptor signaling inhibitors (ARSi) and taxane-based chemotherapy have been established as valid treatment options for patients with mCRPC, by demonstrating a significant survival advantage in phase 3 trials [1]. However, the androgen-receptor plasticity and the adaptive mechanisms of prostate cancer cells eventually produce resistance to therapies and treatment failure [2]. The androgen-receptor isoform encoded by splice variant 7 (AR-V7) lacks the ligand-binding domain, leading to constitutive activation of the androgen receptor, and its detection in circulating tumor cells (CTC) has been associated with resistance to ARSi [3]. 

In 2014, Antonarakis and colleagues investigated the role of AR-V7 in two small cohorts of patients treated with ARSi [3]. The AdnaTest platform for CTC isolation was adapted for detection and quantification of AR-FL and AR-V7 by quantitative real-time PCR using custom primers. None of the 18 men who had detectable AR-V7 in circulating tumor cells (CTC) showed response to treatment with enzalutamide or abiraterone acetate. Conversely, detection of AR-V7 was not associated with primary resistance to taxane chemotherapy in the study on 37 mCRPC patients treated with docetaxel or cabazitaxel [4]. Based on these results, the Johns Hopkins team collected a validation set of 202 patients with mCRPC starting abiraterone or enzalutamide and investigated the prognostic value of CTC and AR-V7 detection [5]. Patients’ outcomes were best for CTC− patients, intermediate for CTC+/AR-V7− patients, and worse for CTC+/AR-V7+ patients. These data led the authors to suggest that the modified-AdnaTest CTC-based AR-V7 mRNA assay should be interpreted using these three separate prognostic categories.

Scher and colleagues reproduced the results of Antonarakis using the Epic Sciences CTC platform that detected the nuclear-localized AR-V7 and analyzed the blood sample of 161 patients with mCRPC [6]. Patients who received ARSi and had basal AR-V7-positive CTC showed worse outcomes in all time-to-event measures, whereas AR-V7 status did not affect the outcome of patients treated with taxanes. In the validation study, Scher and colleagues enrolled 142 patients with mCRPC and tested if AR-V7 status could predict survival according to different treatment received [7]. In the high-risk group including 70 men, patients with AR-V7+ high-risk disease treated with taxanes showed superior overall survival (OS) relative to those treated with ARSi, whereas patients with AR-V7− high-risk disease treated with ARSi had superior OS compared to those treated with taxanes. 

The PROPHECY study investigated the role of AR-V7 in 118 poor-risk men treated with abiraterone or enzalutamide using both the modified-AdnaTest CTC AR-V7 mRNA assay and the Epic Sciences CTC nuclear-specific AR-V7 protein assay [8]. AR-V7 detection by both platforms was associated with shorter progression-free survival (PFS) and OS, with a percentage agreement between the two AR-V7 assays of 82%. Notably, the PROPHECY trial did not include a cohort of patients treated with chemotherapy as comparison, and good-risk patients were not enrolled. Although many other studies support the potentially predictive role of AR-V7, detected in CTC or in tumor tissues [9,10,11,12,13,14,15], no adequately powered prospective validation of AR-V7 in patients treated with ARSi versus chemotherapy is available to date. 

Circulating androgen receptor (AR) copy number variations in plasma DNA have been also associated with worse outcome in mCRPC after treatment with ARSi [16,17,18]. In addition, a recent study has shown that AR gain in plasma DNA from docetaxel-treated mCRPC patients was associated with worse OS, but AR-gained patients seemed to derive greater benefit from treatment with taxanes than with ARSi [19]. These studies support the importance of the AR itself in the resistance processes and its potential predictive value. The John Hopkins team has reported that, in addition to AR-V7, the full-length androgen receptor (AR-FL) mRNA quantification from CTC has prognostic significance in mCRPC patients starting abiraterone and enzalutamide [20]. In their cohort of 202 patients, AR-FL correlated with positive AR-V7 detection and lower biochemical responses and was associated with worse PFS and shorter OS. Del Re and colleagues have recently published an analysis of AR-FL and AR-V7 from exosomes of patients with mCRPC [21]. They found an association between AR-V7 and AR-FL with PFS and OS. Based on these data, they have proposed that AR-FL could be considered as a predictor biomarker in combination with AR-V7. This figure is quite similar to the categories proposed by Antonarakis and colleagues [5], based on CTC and AR-V7 status. 

Despite these encouraging results, the predictive versus prognostic role of these variables has not been completely established yet and their significance as predictor of response is currently uncertain.

This prospective study aimed to explore the prevalence and significance of CTC, AR-FL and AR-V7 in a cohort of patients with mCRPC. 

## 2. Materials and Methods

### 2.1. Patient Selection

Ethics approval and consent to participate: informed written consent to blood collection and use for experimental purposes was obtained by all patients, following study-protocol approval by the local Ethical Committee (P.R.505REG2015).

Patients with mCRPC referred to our Institution, who were starting a first-line treatment for mCRPC (1L subgroup) or who had been already treated with at least two lines for mCRPC (>2L subgroup), including at least one ARSi and one chemotherapy regimen, were invited to participate in this study. After informed consent, patients underwent a blood sample collection and were prospectively followed with PSA assessments every 4–6 weeks, until death or the limit date of 31 December 2018.

### 2.2. CTC Capture and AR-FL/AR-V7 Analysis

To evaluate AR-FL and AR-V7 on CTC, 5 ml of blood was collected before starting a new line of therapy into dedicated test tube AdnaCollect (Qiagen, Hilden, Germany) and processed within 2 hours as described in the study published by Antonarakis and colleagues [3]. Briefly, CTC were isolated by immuno-magnetic beads with an EpCAM-based method (AdnaTest Prostate Cancer Select), mRNA was obtained and retro-transcribed using the Adna Test Prostate Cancer Detect and SensiScript RT kits (Qiagen, Hilden, Germany), respectively. cDNA was used for AR-FL and AR-V7 detection by a dedicated kit developed by Bird LTD company laboratories (Rezzato, Italy) [22]. CTC positivity was carried out by multiplex PCR reaction using primer specific for prostate-specific antigen (PSA), prostate-specific membrane antigen (PSMA), epidermal growth factor receptor (EGFR), and actin as internal PCR control. PCR products were analyzed on Agilent chip by Bioanalyzer electrophoresis (Agilent Technologies, Santa Clara, CA, USA). Given the intrinsic limits related to the methods for CTC isolation and RNA detection, patients with detectable (below 10 copies/mL), but not quantifiable, AR-V7 and AR-FL on CTC were considered as positive.

### 2.3. Statistical Analysis

The statistical analysis was conducted using the chi-square test for categorical variables and Fisher Exact test when appropriate. Pearson’s correlation was used to test correlations among variables. PSA50 was the endpoint chosen for the analysis of biochemical response and was defined as a decline of at least 50% in PSA values from treatment start. OS was defined as the time elapsed from blood collection date and the date of death for any cause. OS curves were constructed according to the Kaplan–Meier method and compared using the log-rank test [23]. The Cox proportional model hazard ratios (HR) estimates and their 95% confidence intervals (CI) were also calculated. Only variables with a *p* value < 0.05 at univariable analysis were included in the multivariable models. The performance of the models was measured using the concordance *C*-index [24]. All *p* values were two-tailed. The IBM software Statistical Package for Social Sciences (SPSS) version 25.0 for Windows (SPSS Inc., Chicago, IL, USA) and the Software for Statistics and Data Science (STATA) version 11 were used for data analysis. Given the small sample size and the exploratory purpose of this study, adjustments for multiplicity were not performed.

## 3. Results

### 3.1. Patients Cohort

Thirty-nine patients with mCRPC who met the inclusion criteria were enrolled in this study, of them 24 were included in the 1L subgroup and 15 in the >2L subgroup. After sample collection, 1L patients received abiraterone acetate, enzalutamide, or docetaxel, whereas treatments of >2L subgroup included abiraterone acetate, cabazitaxel, docetaxel, cyclophosphamide, enzalutamide, mitoxantrone, and vinorelbine. Drug dosages and schedules were those commonly used in clinical practice, according to physician choice and international guidelines on prostate cancer [25]. The main characteristics of cohort patients are summarized in Table 1 and Supplementary Table S1.

### 3.2. CTC Isolation and AR-FL/AR-V7 Prevalence

CTC were identified in 21 of the 39 blood samples (53.8%) (Table 1). A higher percentage of CTC+ patients was found in the >2L subgroup than in the 1L subgroup (11/15, 73.3% vs. 10/24, 41.7%; *p* = 0.054). AR-FL mRNA was detected in 15/21 of CTC+ patients (71.4%); seven patients had less than 10 copies/mL and eight patients had ≥10 copies/mL; irrespective of the number of AR-FL copies, a higher percentage of AR-FL+ patients was found in the >2L subgroup than in the 1L subgroup (9/15, 60% vs. 6/24, 25%; *p* = 0.03). Overall, AR-V7 expression was detected in seven of 39 patients (17.9%); 2/24 patients (8.3%) were AR-V7 positive in the 1L subgroup and 5/15 patients (33.3%) were AR-V7 positive in the >2L subgroup (*p* = 0.08). None of our patients had detectable EGFR. AR-V7 correlated significantly with both AR-FL copies (*r* = 0.59 *p* < 0.001) and CTC detection (*r* = 0.43 *p* = 0.006).

### 3.3. Association of CTC, AR-FL, and AR-V7 with PSA50 Response 

**Figure 1:**
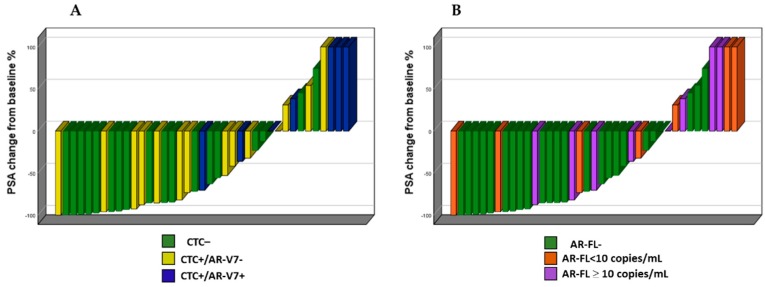
Best prostate-specific antigen (PSA) response according to circulating tumor cells/androgen receptor splice variant 7 (CTC/AR-V7) status (**A**) and number of androgen receptor full-length (AR-FL) copies (**B**).

**Figure 2:**
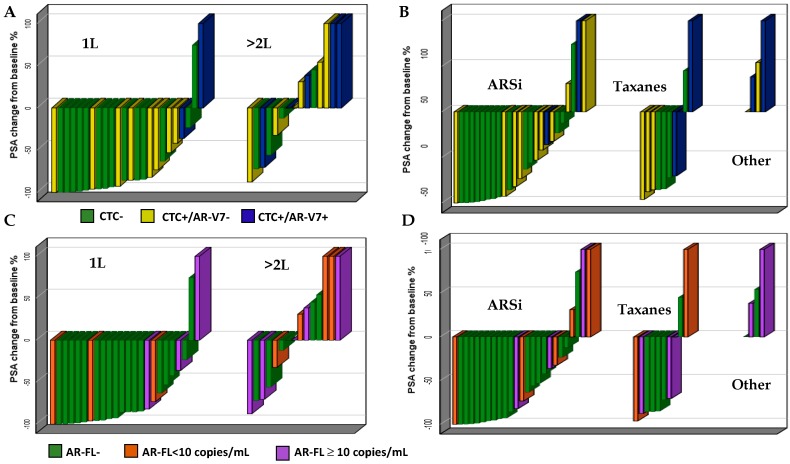
Subgroup analysis. Best PSA response according to CTC and AR-V7 by treatment line (**A**) and type of treatment (**B**) and best PSA response according to AR-FL copies by treatment line (**C**) and type of treatment (**D**).

A higher percentage of PSA50 responses was observed in CTC− than in CTC+ patients (14/18, 77.8% vs. 9/21, 42.9%; *p* = 0.03) (Figure 1A). Only one of seven AR-V7+ patients (14.3%) reached the PSA50, compared to 22/32 (68.8%) of AR-V7 negative patients (*p* = 0.008). Irrespective of treatment line, 5/11 (45%) patients who did not show any PSA response to therapies were AR-V7 positive. A higher percentage of biochemical responses were also observed in AR-FL negative compared to AR-FL positive patients (17/24, 70.8% vs. 6/15, 40%; *p* = 0.057); however, three of seven patients (42.8%) with the highest number of AR-FL copies (≥10 copies/mL) reached the PSA50 (Figure 1B); two of them received taxanes and one received ARSi (Figure 2D).

Patients included in the 1L subgroup showed a higher number of PSA50 responses than patients who started a >2L treatment. PSA50 was reached in 19/24 (79.2%) patients included in the 1L cohort, compared to 4/15 (26.7%) patients included in the >2L subgroup (Figure 2A–C).

Neither of the two AR-V7+ patients treated with ARSi reached the PSA50, whereas one AR-V7+ patient showed PSA50 response to third-line cabazitaxel. Of the four highly-pretreated patients who received palliative treatments (cyclophosphamide, mitoxantrone, vinorelbine), none showed a PSA response (all were CTC positive and two were AR-V7 positive) (Figure 2B).

### 3.4. Association of CTC, AR-FL, and AR-V7 with OS

**Figure 3:**
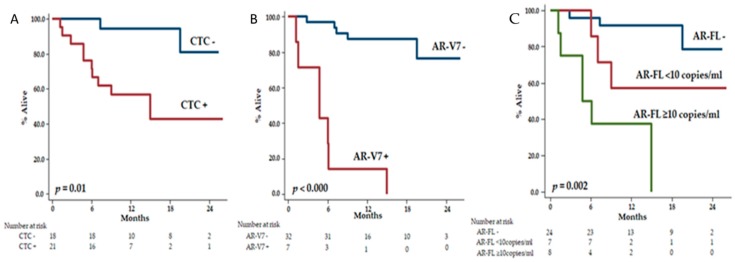
Overall survival according to CTC (**A**), AR-V7 (**B**) and AR-FL (**C**).

We performed univariable analysis for significant prognostic variables (*p* < 0.05), and we found that median PSA value, treatment line at study entry, number of metastatic sites, CTC status, AR-FL status, and AR-V7 status were all associated with OS (Supplementary Table S2). In the Kaplan–Meier estimations, the median OS of CTC+ patients was 15.0 months (95% CI 2.8–27.2) and was not reached in CTC− patients (HR: 7.40, 95% CI 1.53–35.82; *p* = 0.01) (Figure 3A). The estimations for OS were 4.7 months (95% CI 0.6–8.9) in AR-V7+ patients and not reached in AR-V7− patients (HR: 20.31, 95% CI 5.54–74.49; *p* < 0.000) (Figure 3B). Patients with ≥10 copies/mL of AR-FL showed the estimated shortest OS (4.7 months (95% CI 0.5–9.0), followed by patients with a low number of detectable AR-FL copies (<10 copies/mL) (not reached) and by patients without detection of AR-FL, who showed the longest OS (not reached, overall *p* = 0.002) (Figure 3C). AR-V7 was the only variable that retained the statistical significance in the Cox multivariable model (*Model 1*, AR-V7+: HR 6.09, 95% CI 1.53–24.31; *p* = 0.01) with a *C*-index of 0.92 (0.86–0.98) (Table 2).

### 3.5. Exploratory Prognostic Nomograms for OS

**Figure 4:**
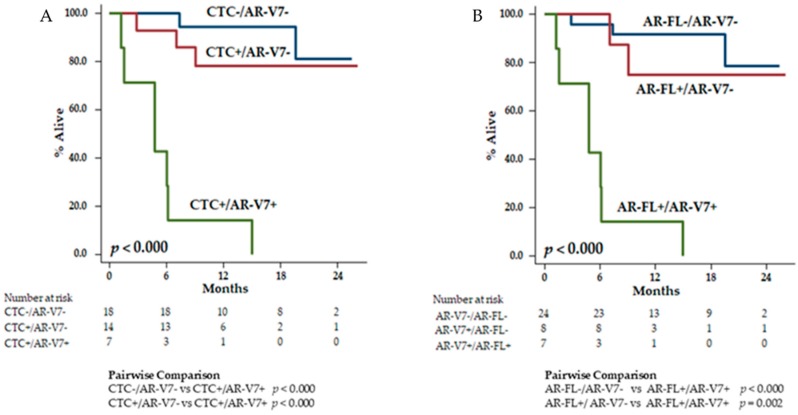
Prognostic nomograms according to combinations of CTC with AR-V7 (**A**) and AR-FL with AR-V7 (**B**).

Consistently with literature reports [5,21], we elaborated two prognostic nomograms including CTC/AR-V7 (Figure 4A) and AR-FL/AR-V7 (Figure 4B). In the Cox analysis, the two models did not outperform the prognostic model that included AR-V7 alone. The *C*-indexes were 0.91 (0.84–0.96) for AR-V7/CTC, 0.90 (0.83–0.97) for AR-V7/AR-FL, and 0.92 (0.86–0.98) for AR-V7 alone.

## 4. Discussion

In the present study, we used a modified-AdnaTest CTC-based AR-FL and AR-V7 mRNA assay with custom primers [22], similar to that used by Antonarakis and colleagues [3], to assess the prognostic and predictive significance of CTC, AR-FL, and AR-V7 in a monocentric prospective cohort of patients with mCRPC at different phases of disease. 

In our exploratory cohort, we observed that CTC, AR-FL, and AR-V7 were all enriched in pretreated mCRPC patients compared to those starting a first-line regimen (Table 1). This figure is well known in respect to AR-V7 [5,13], but less information is currently available regarding AR-FL in CTC [20]. 

Overall, a lower percentage of biochemical responses was observed in the AR-V7+ patients than in AR-V7− (Figure 1 and Figure 2). Neither of the two AR-V7+ patients treated with ARSi showed a biochemical response. Notably, a higher percentage of biochemical responses were also observed in CTC− compared to CTC+ patients and in AR-FL- compared to AR-FL+ patients. Despite our results potentially being biased due to the small sample size and heterogeneity of the cohort, these observations are consistent with the literature data [3,4,5,6,7,8,9,10,11,12,13,14,15]. Among patients with >10 copies/mL of AR-FL, 2/2 treated with taxane reached the PSA50 compared to 1/3 treated with ARSi. Although few patients are considered, this observation might be consistent with a possible positive effect of taxanes in patients with AR gain [19]. 

To our knowledge, this is the first report that included a group of highly pretreated patients receiving palliative therapies. This population well represents a condition of intensive selective pressure from treatments (ARSi and chemotherapy). Of the four patients treated with cyclophosphamide, mitoxantrone, and vinorelbine, none showed a PSA response (all were CTC positive and two were AR-V7 positive). This observation suggests that such treatments might not able to overcome the resistance induced by prior therapies and confirms the strong enrichment of AR-V7 and CTC in end-stage populations.

AR-V7, AR-FL, and CTC were all identified as prognostic variables for OS in univariable analysis (Supplementary Table S2), but AR-V7 was the only factor that retained the statistical significance in the Cox multivariable model (Table 2). 

In our exploratory prognostic nomograms, we did not find that the combination of AR-V7 with CTC or with AR-FL could better predict for patients’ prognosis compared to AR-V7 alone (Figure 3 and Figure 4). This result needs confirmation in a larger cohort and might be the result of a small sample size.

We also acknowledge that the significance of CTC, AR-FL, and AR-V7 in our prospective series might be at least partly amplified by the method for CTC detection and heterogeneity of the cohort. Notably, AR-V7 correlated with both AR-FL and CTC detection. There is a higher probability of detecting CTC, and in turn AR-FL/AR-V7, in a population with more advanced and incurable disease stage, because of the higher disease burden and higher number of CTC. This figure is confirmed by the highest PSA values that were observed in CTC+ AR-V7+ patients, followed by CTC+ AR-V7− and by CTC− AR-V7− patients (Table 1). Similarly, a negative AR-V7 status does not necessarily imply that AR-V7 is not playing a role in the resistance processes. In fact, the detection of AR-V7 is subordinated to the challenging detection of CTC, which might be rare in patients with low disease burden or low tumor shrinkage. This notion is confirmed by a recent study performed by Sharp and colleagues [13]. The authors analyzed 181 mCRPC patients and did not find a significant difference in OS between CTC+/AR-V7+ and CTC+/AR-V7−patients when adjusting for baseline characteristic including CTC count. They also demonstrated that AR-V7 protein expression in mCRPC biopsies is often not consistent with the result on CTC. False positives and false negatives can derive from the limits of CTC isolation and AR-V7 analysis or can be due to intrapatient tumor sampling variability. Finally, although AR-V7 seems to predict for response to ARSi, this androgen-receptor variant does not explain all of the mechanisms of resistance, since some AR-V7+ patients still respond to ARSi therapy, and some AR-V7+ patients do not respond to chemotherapy [26]. This observation is confirmed in our small cohort of patients, in which three AR-V7 negative patients did not respond to first-line ARSi, and many AR-V7+ patients did not show response to chemotherapy. 

## 5. Conclusions

Our study confirms the prognostic role of AR-V7, whose detection might be relevant during the treatment choices for patients with mCRPC. AR-V7 seems to be the most promising biomarker to predict for response to ARSi, however a prospective validation study in chemotherapy-treated versus ARSi-treated patients is still needed to implement this test in clinical practice. In our small cohort, the combination of AR-V7 with CTC or with AR-FL did not outperform the prognostic model including only AR-V7. Further studies are strongly warranted to assess the role of these biomarkers in patients with mCRPC.

## Figures and Tables

**Table 1:** Patient characteristics.

Variable	CTC−/AR-V7−	CTC+/AR-V7−	CTC+/AR-V7+	All
	*n* = 18	*n* = 14	*n* = 7	*n* = 39
Age				
Median (range), years	74 (56–84)	72 (58–84)	70 (66–84)	72 (56–84)
PSA				
Median (range), ng/mL	11.8 (2.93–516.9)	63.9 (0.33–564.9)	514.0 (35.22–4688)	35.2 (0.33–4688)
LDH				
Median (range), U/L	216 (138–301)	234 (152–339)	452 (121–1616)	228 (121–1616)
Planned treatment line at study entry *				
1L	14 (77.8%)	8 (57.1%)	2 (28.6%)	24 (61.5%)
>2L	4 (22.2%)	6 (42.9%)	5 (71.4%)	15 (38.5%)
Treatment at AR-V7 sample ^Δ^				
ARSi Therapy	14 (77.8%)	9 (64.3%)	2 (28.6%)	25 (64.1%)
Cabazitaxel/Docetaxel	4 (22.2%)	3 (21.4%)	3 (42.8%)	10 (25.6%)
Other	-	2 (14.3%)	2 (28.6%)	4 (10.3%)
Bone metastases				
Absent	6 (33.3%)	2 (14.3%)	-	8 (20.5%)
Present	12 (66.7%)	12 (85.7%)	7 (100.0%)	31 (79.5%)
Visceral metastases				
Absent	16 (88.9%)	10 (71.4%)	6 (85.7%)	32 (82.1%)
Present	2 (11.1%)	4 (28.6%)	1 (14.3%)	7 (17.9%)
Number of metastatic sites				
=1 site	12 (66.7%)	7 (50.0%)	2 (28.6%)	21 (53.8%)
>1 sites	6 (33.3%)	7 (50.0%)	5 (71.4%)	18 (46.2%)

* 1L = first-line therapy for metastatic castration-resistant prostate cancer (mCRPC); >2L = third-line or more for mCRPC. Δ ARSi therapy = androgen-receptor signaling inhibitors (abiraterone acetate, enzalutamide); Other = cyclophosphamide, mitoxantrone, vinorelbine.

**Table 2:** Multivariable Analysis.

Variable	1° Model		2° Model	3° Model			4° Model	
	HR (95% CI)	*p*≤	HR (95% CI)	*p*≤	HR (95% CI)	*p*≤	HR (95% CI)	*p*≤
**PSA (ng/mL)**								
<35 ng/mL	1		1		1		1	
≥35 ng/mL	6.63 (0.52–84.11)	0.1	7.31 (0.54–98.24)	0.1	6.47 (0.49–85.59)	0.1	5.47 (0.38–78.17)	0.2
Treatment line *								
1L	1		1		1		1	
>2L	2.54 (0.53–12.08)	0.2	4.03 (0.95–17.14)	0.06	3.17 (0.70–14.35)	0.1	2.21 (0.44–11.19)	0.3
**Metastatic sites**								
=1 site	1		1		1		1	
>1 sites	1.56 (0.36–6.93)	0.5	1.66 (0.38–7.21)	0.5	1.93 (0.45–8.33)	0.4	2.15 (0.39–12.20)	0.4
**AR-V7**								
Negative	1						1	
Positive	6.09 (1.53–24.31)	0.01	−	−	−	−	8.96 (0.94–85.38)	0.057
**CTC**								
Negative			1				1	
Positive	−	−	2.85 (0.42–19.18)	0.3	−	−	1.08 (0.07–16.94)	0.9
**AR-FL**								
Negative					1	0.2	1	0.6
<10 copies/mL	−	−	−	−	2.80 (0.48–16.36)	0.2	1.91 (0.15–23.76)	0.6
≥10 copies/mL					4.16 (0.83–20.89)	0.08	0.73 (0.04–14.33)	0.8
***C*-Index**	0.92 (0.86–0.98)		0.86 (0.77–0.94)		0.87 (0.78–0.95)		0.91 (0.85–0.98)	

* 1L = first-line therapy for metastatic castration-resistant prostate cancer (mCRPC); >2L = third-line or more for mCRPC.

## Supplementary Materials

The following are available online at https://www.mdpi.com/2072-6694/11/9/1365/s1, Table S1: Patient Characteristics according to Treatment Line at Study Entry (1L vs. >2L), Table S2: Univariate Analysis, Table S3: Results of biomarkers’ analysis for each patient included in the study.
