# Supplementary material for: Role of Circulating Tumor Cells (CTC), Androgen Receptor Full Length (AR-FL) and Androgen Receptor Splice Variant 7 (AR-V7) in a Prospective Cohort of Castration-Resistant Metastatic Prostate Cancer Patients
Source: Cancers (Basel). 2019 Sep 13;11(9):1365. doi: 10.3390/cancers11091365 (PMC6770005; doi:10.3390/cancers11091365)
Supplement: Supplementary file 1 [file cancers-11-01365-s001.pdf]

Supplementary Materials: Role of Circulating Tumor Cells (CTC), Androgen Receptor Full Length (AR-FL) and Androgen Receptor Splice Variant 7 (AR-V7) in a Prospective Cohort of Castration-Resistant Metastatic Prostate Cancer Patients.

Carlo Cattrini, Alessandra Rubagotti, Linda Zinoli, Luigi Cerbone, Elisa Zanardi, Matteo Capaia, Paola Barboro and Francesco Boccardo

Table S1. Patient Characteristics according to Treatment Line at Study Entry (1L vs >2L).

Variable	1L (*n* = 24)	>2L (*n* = 15)
Age		
Median (range), years	75.5 (56–84)	70.0 (58–84)
PSA		
Median (range), ng/mL	13.36 (0.33–564.90)	90.47 (3.99–4688.00)
LDH		
Median (range), U/L	220 (138–628)	229 (121–1616)
Treatment at AR-V7 sampleΔ		
ARSi Therapy	20 (83.3%)	5 (33.3%)
Cabazitaxel/Docetaxel	4 (16.7%)	6 (40.0%)
Other	-	4 (26.7%)
Bone metastases		
Absent	7 (29.2%)	1 (6.7%)
Present	17 (70.8%)	14 (93.3%)
Visceral metastases		
Absent	21 (87.5%)	11 (73.3%)
Present	3 (12.5%)	4 (26.7%)
Number of metastatic sites		
=1 site	14 (58.3%)	7 (46.7%)
>1 sites	10 (41.7%)	8 (53.3%)

Δ ARSi therapy= Androgen-Receptor Signaling inhibitors (abiraterone acetate, enzalutamide); Other= cyclophosphamide, mitoxantrone, vinorelbine.

Table S2. Univariate Analysis.

Variable	HR (95% CI)	*p*≤
Age, years		
≤72	1	
>72	1.21 (0.39–3.77)	0.7
PSA (ng/mL)		
<35 ng/mL	1	
≥35 ng/mL	19.77 (2.43–160.53)	0.005
LDH, U/L		
≤225 U/L	1	
>225 U/L	2.02 (0.59–6.89)	0.2
Treatment line*		
1L	1	
>2L	7.41 (1.94–28.24)	0.003
Bone metastases		
Absent	1	
Present	3.64 (0.47–28.35)	0.2
Visceral metastases		
Absent	1	
Present	1.73 (0.46–6.42)	0.4
Number of metastatic sites		
=1 site	1	
>1 sites	4.71 (1.26–17.57)	0.02
AR_V7		
Negative	1	

Positive	20.31 (5.54–74.48)	0.000
CTC		
Negative	1	
Positive	7.40 (1.53–35.82)	0.01
AR-FL		
Negative	1	0.002
<10 copies/mL	4.45 (0.88–22.39)	0.07
≥10 copies/mL	13.52 (3.11–58.85)	0.001

*1L = First-line therapy for metastatic castration-resistant prostate cancer (mCRPC); >2L = Third-line or more for mCRPC.Δ ARSi therapy = Androgen-Receptor Signaling inhibitors (abiraterone acetate, enzalutamide); Other = cyclophosphamide, mitoxantrone, vinorelbine.

Table S3. Results of Biomarkers' Analysis for Each Patient Included in the Study.

Treatment Line	PSA	LDH	CTC	PSMA	PSA	AR-V7	AR-FL	ARV7/ARFL Ratio	PSA50
1L	8.1	237	POS	NEG	NEG	NEG	POS (< 10 cp/mL)		YES
1L	30.8	218	NEG	NEG	NEG	NEG	NEG		NO
1L	13.5	NA	NEG	NEG	NEG	NEG	NEG		YES
1L	141.5	138	NEG	NEG	NEG	NEG	NEG		YES
1L	564.9	172	POS	NEG	POS	NEG	NEG		YES
1L	13.2	214	NEG	NEG	NEG	NEG	NEG		YES
1L	2.9	188	NEG	NEG	NEG	NEG	NEG		YES
1L	12.8	199	NEG	NEG	NEG	NEG	NEG		YES
1L	10.8	207	NEG	NEG	NEG	NEG	NEG		YES
1L	35.2	216	POS	NEG	POS	POS (29.4 cp/mL)	POS (330.0 cp/mL)	8.9%	NO
1L	97.7	249	POS	NEG	NEG	NEG	POS (85.0 cp/mL)		YES
1L	7.6	234	NEG	NEG	NEG	NEG	NEG		YES
1L	29.9	301	NEG	NEG	NEG	NEG	NEG		YES
1L	3.1	203	NEG	NEG	NEG	NEG	NEG		YES
1L	87.8	248	POS	NEG	POS	NEG	NEG		YES
1L	51.5	170	POS	NEG	NEG	NEG	POS (< 10 cp/mL)		YES
1L	6.9	203	NEG	NEG	NEG	NEG	NEG		YES
1L	9.0	230	NEG	NEG	NEG	NEG	NEG		YES
1L	0.3	220	POS	NEG	NEG	NEG	POS (< 10 cp/mL)		YES
1L	25.6	228	NEG	NEG	NEG	NEG	NEG		NO
1L	7.3	235	NEG	NEG	NEG	NEG	NEG		YES
1L	47.9	241	POS	NEG	POS	NEG	NEG		YES
1L	140.0	628	POS	POS	POS	POS (< 10 cp/mL)	POS (29.8 cp/mL)		NO
1L	3.6	234	POS	POS	POS	NEG	NEG		NO
> 2L	514.0	229	POS	NEG	POS	POS (17.2 cp/mL)	POS (46.7 cp/mL)	36.8%	YES
> 2L	4.0	208	NEG	NEG	NEG	NEG	NEG		YES
> 2L	59.5	NA	NEG	NEG	NEG	NEG	NEG		YES
> 2L	24.2	152	POS	NEG	NEG	NEG	POS (< 10 cp/mL)		NO
> 2L	550.2	339	POS	NEG	NEG	NEG	POS (18.0 cp/mL)		YES
> 2L	6.0	275	NEG	NEG	NEG	NEG	NEG		NO
> 2L	516.9	228	NEG	NEG	NEG	NEG	NEG		NO
> 2L	1036.0	839	POS	POS	NEG	POS (<10 cp/mL)	POS (33.9 cp/mL)		NO
> 2L	4688.0	452	POS	POS	POS	POS (11.6 cp/mL)	POS (17.8 cp/mL)	65.2%	NO
> 2L	67.0	271	POS	POS	POS	NEG	POS (< 10 cp/mL)		NO
> 2L	78.1	121	POS	POS	POS	POS (<10 cp/mL)	POS (< 10 cp/mL)		NO
> 2L	97.9	NA	POS	POS	POS	NEG	POS (< 10 cp/mL)		NO
> 2L	958.6	1616	POS	POS	POS	POS (257 cp/mL)	POS (193.0 cp/mL)	133.2%	NO
> 2L	60.9	213	POS	POS	POS	NEG	NEG		NO
> 2L	90.5	186	POS	POS	POS	NEG	NEG		NO

© 2019 by the authors. Licensee MDPI, Basel, Switzerland. This article is an open access article distributed under the terms and conditions of the Creative Commons Attribution (CC BY) license (http://creativecommons.org/licenses/by/4.0/).
